# Supplementary figures and images for: Analysis of Human and Microbial Salivary Proteomes in Children Offers Insights on the Molecular Pathogenesis of Molar-Incisor Hypomineralization
Source: Biomedicines. 2022 Aug 24;10(9):2061. doi: 10.3390/biomedicines10092061 (PMC9495719; doi:10.3390/biomedicines10092061)

Taxonomy profile for BuiltInNEW.megan (rank=Genus)

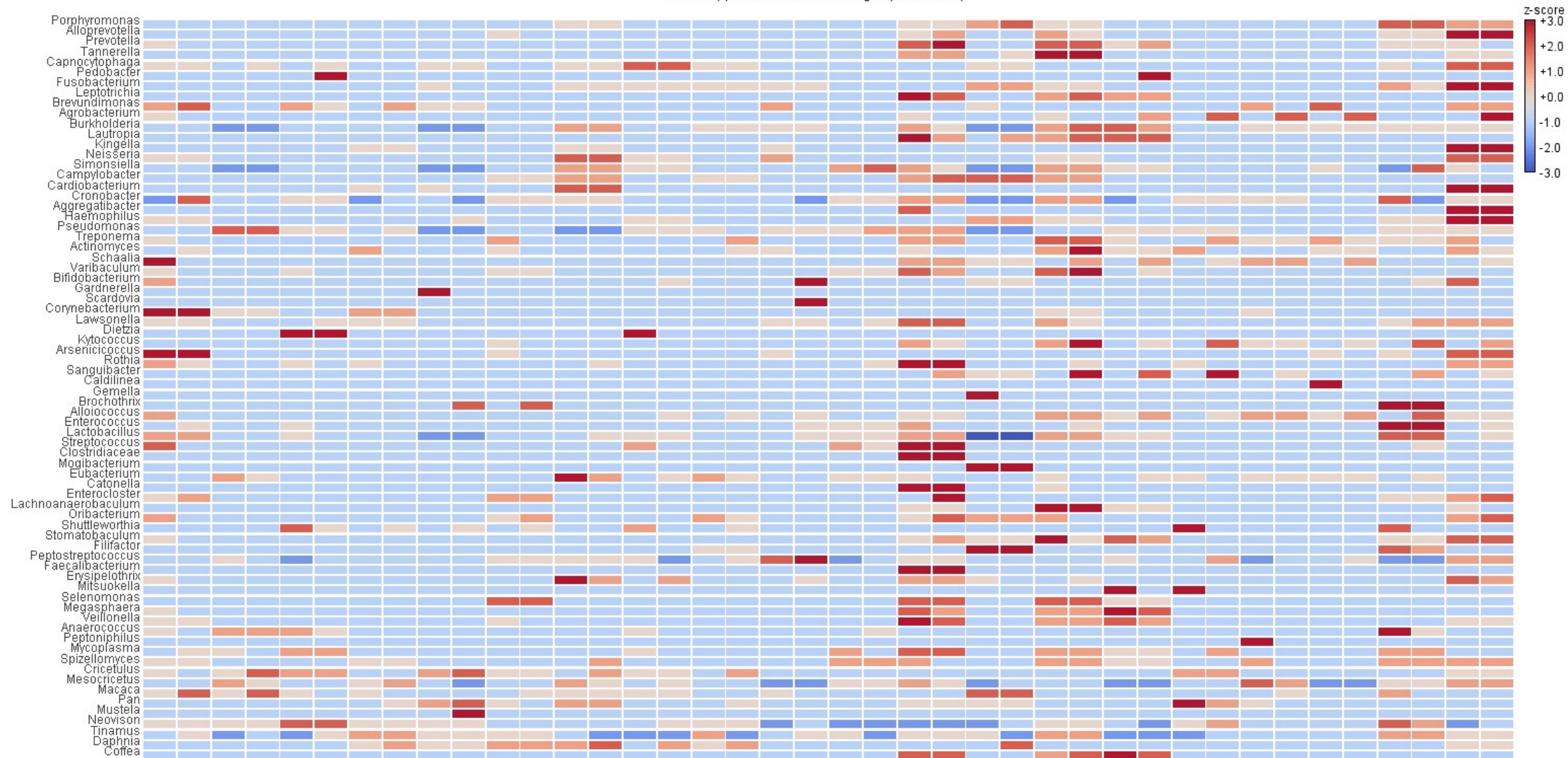

Samples

MIH

HEALTHY CONTROLS

Supplement: Supplementary file 1 [file biomedicines-10-02061-s001.zip › SUPPL FIG S1.pdf]
